# Microbiota and Phage Therapy: Future Challenges in Medicine

**DOI:** 10.3390/medsci6040086

**Published:** 2018-10-05

**Authors:** Armelle Paule, Domenico Frezza, Marvin Edeas

**Affiliations:** 1 International Society of Microbiota, Tokyo 104-0032, Japan; paule@microbiota-site.com; 2 University of Roma Tor Vergata, 00118 Roma, Italy; frezza@uniroma2.it; 3 Cochin Institute—INSERM U1016, University Paris Descartes, Development, Reproduction and Cancer, Cochin Hospital, 75014 Paris, France

**Keywords:** phage therapy, microbiota, chronic disease, pathology treatment, mitochondria

## Abstract

An imbalance of bacterial quantity and quality of gut microbiota has been linked to several pathologies. New strategies of microbiota manipulation have been developed such as fecal microbiota transplantation (FMT); the use of pre/probiotics; an appropriate diet; and phage therapy. The presence of bacteriophages has been largely underestimated and their presence is a relevant component for the microbiome equilibrium. As a promising treatment, phage therapy has been extensively used in Eastern Europe to reduce pathogenic bacteria and has arisen as a new method to modulate microbiota diversity. Phages have been selected and “trained” to infect a wide spectrum of bacteria or tailored to infect specific antibiotic resistant bacteria present in patients. The new development of genetically modified phages may be an efficient tool to treat the gut microbiota dysbiosis associated with different pathologies and increased production of bacterial metabolites and subsequently decrease systemic low-grade chronic inflammation associated with chronic diseases. Microbiota quality and mitochondria dynamics can be remodulated and manipulated by phages to restore the equilibrium and homeostasis of the system. Our aim is to highlight the great interest for phages not only to eliminate and control pathogenic bacterial infection but also in the near future to modulate the microbiota by adding new functions to selected bacteria species and rebalance the dynamic among phages and bacteria. The challenge for the medicine of tomorrow is to re-think and redesign strategies differently and far from our traditional thinking.

## 1. Manipulation of Microbiota Diversity: Future Challenge in Medicine

The gut microbiota plays a crucial role in the host’s metabolism, physiology and health [1,2]. It protects the host against pathogenic infections, stimulates the immune system, and regulates oxidative stress [3]. There is strong evidence that gut microorganisms are involved in many chronic diseases and have an important impact on the homeostasis of the gut itself and may also affect the immune states of extraintestinal organs including the liver, kidney, cardiovascular and bone systems [4,5]. Moreover, studies have shown the possible relationship between the composition of gut microbiota and the brain via metabolites produced by bacteria which act as signal-mediators [6,7]. One example is butyrate, a short chain fatty acid (SCFA) produced by the bacteria during the fermentation of fiber, which may improve brain health [8].

**Figure 1:**
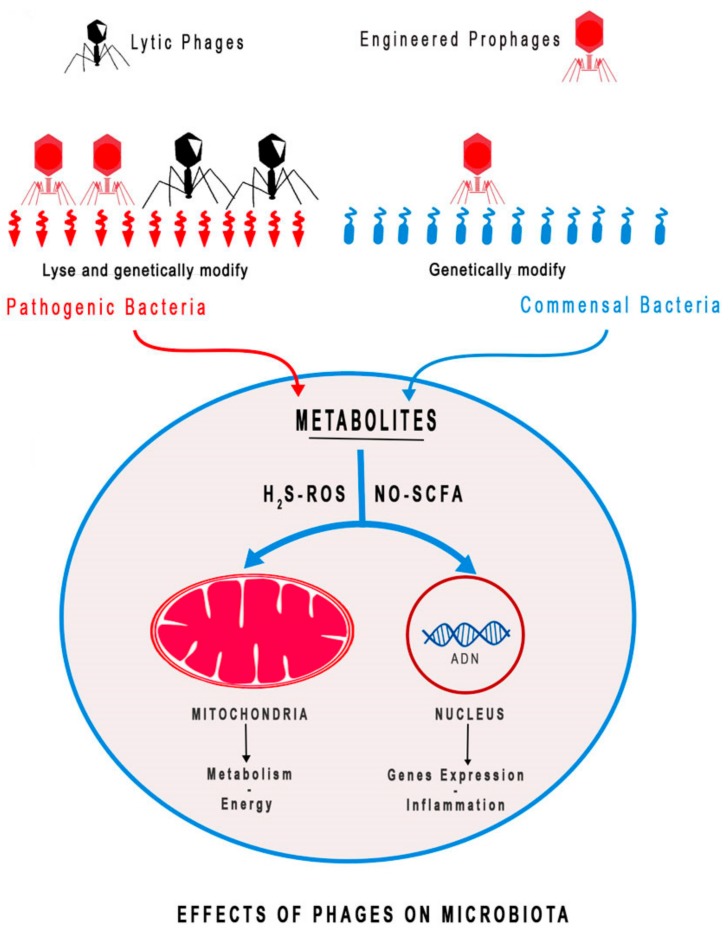
Bacteriophages modulate microbiota quality and quantity. Gut microbiota release metabolites and factors including hydrogen sulfide (H2S), reactive oxygen species (ROS), nitric oxide (NO) and short chain fatty acids (SCFAs) that can directly affect mitochondria activity, adenosine triphosphate (ATP) production, and nuclear genome. High ROS production can trigger an inflammatory response and increase cell oxidative stress. Furthermore, cell stress can trigger mitochondrial and bacterial DNA insertion in the nuclear genome leading of cellular gene expression. Nitric oxide can inhibit the tricarboxylic acid cycle (TCA) by reducing acetyl-CoA production. In addition, high production of hydrogen sulfide (H2S) by the microbiota inhibit complex IV of the electron transfer chain (ETC). SCFAs, in particular butyrate, are able to fuel the TCA cycle. In parallel, SCFAs can induce release of anti-inflammatory IL-10 cytokines and signaling hormone GLP-1 to reduce energy intake. Unbalanced microbiota displayed low bacterial diversity and potentially increased the proportion of pathogenic bacteria that favor mucosal inflammation. Manipulation of microbiota by lytic phage can be used to selectively reduce pathogenic bacteria. In addition, prophages that carry biosynthesis genes of metabolites that positively regulate mucosal inflammation can be engineered to genetically modify commensal bacteria [9,10].

As illustrated in Figure 1, we recently highlighted that the microbiota releases metabolites such as hydrogen sulfide (H2S), nitric acid (NO), and SCFAs that can interfere with the mitochondrial respiratory chain and adenosine triphosphate (ATP) production and affect gene expression in particular inflammatory cytokines [9]. The amount of metabolites such as NO and H2S can affect mitochondrial metabolism and energy dynamics. Both are able to inhibit the tricarboxylic acid cycle (TCA) by reducing acetyl-CoA production. In addition, high production of H2S by the microbiota inhibit complex IV of the electron transfer chain (ETC). In parallel, SCFAs, in particular butyrate, are able to fuel the TCA cycle [9,10].

Moreover, our recent clinical study revealed for the first time a connection between the occurrence of cramps in hemodialysis patients and a dysbiosis of microbiota and a dysfunction of mitochondria [10]. Indeed, in patients with cramps, gut microbiota diversity seemed lower and some genera including *Helicobacter*, *Lachnospira*, *Roseburia*, and *Haemophilus* seemed overexpressed. Also, a significant increase of citratemia and significant lowering mitochondrial function were observed. 

A decrease in the bacterial diversity in the microbiota favors the recognition of pathogenic bacteria by the Pattern Recognition Receptor (PRR) system [9]. This recognition is associated with an increase of the cellular oxidative stress level and the subsequent relocalization of Nuclear Factor Kappa B (NF-κB) in the nucleus to activate the transcription of numerous proinflammatory factors. Chronic diseases, including obesity, type 2 diabetes, atherosclerosis, Crohn’s disease and some cancers, are linked with low-grade chronic inflammation. This low-grade inflammation triggers the production of proinflammatory markers, which in particular is due to the high transcriptional activity of NF-κB. Based on this mechanism, restoring the balance of gut microbiota should decrease low-grade chronic inflammation and oxidative stress observed in chronic disease patients. Additionally, the leaky gut syndrome should be taken into consideration as observed in Alzheimer’s [11] and Parkinson’s [12] diseases. 

Many strategies have been developed to modulate the microbiota. Transfer of the microbiota isolated from feces of obese or lean mice into germ-free mice showed that animals receiving the microbiota from obese mice presented a weight gain compared to the ones receiving lean mouse microbiota [13]. This first study published on fecal transplantation showed that manipulation of quality and quantity of the microbiota could be used to treat chronic diseases. In humans, fecal transplantation therapy (FMT) has been used with success to treat infection by the antibiotic resistant bacteria, *Clostridium difficile* [14,15,16] or to increase insulin sensitivity of patients [17]. Recently, microbiota transfer therapy has been observed to improve both gastrointestinal and autism symptoms of autism spectrum disorders diagnosed children [18]. Additionally, a study observed seizure protection in mice after transplantation of a ketogenic diet gut microbiota [19]. However, many problems may arise with this therapy since it can increase the risks of transmitting other pathogens, although the origin of transplants are from healthy subjects with their microbiome in equilibrium [20]. 

Alternatively, the use of probiotics, defined as living microorganisms that improve the imbalance of the gut microbiota, has been explored. The most common strains used as probiotics, are members of *Lactobacilli*, *Enterococci* and *Bifidobacteria* groups [21]. Such therapy also shows significant success for the treatment of inflammatory bowel disease [22,23]. However, the probiotic products on the market need more studies in terms of quality and safety. 

Since the release of metabolites by microbiota depends on the diet of the subjects and the composition of the microbiota, an appropriate diet is important to regulate microbiota activity, as observed with butyrate produced by a diet enriched in fiber, which can affect neurodegeneration and promote regeneration [8].

A third promising alternative could be the use of bacteriophages, also defined as natural born killers During the 5th World Congress on Targeting Phage and Antibiotic Resistance [24], we dedicated a full session to microbiota and phages. Among the topics covered, the strategic role of phage therapy to modulate the microbiota was discussed. 

Indeed, there is a constant input of bacteriophages in the gut due to the food consumption but the effect of bacteriophages on the microbiota balance is poorly known [25]. The influence of bacteriophages must be considered as the most important factor, which controls the gut microbiota and should be explored. 

The use of phages as therapy has widely been discussed in Europe by the European Medicines Agency for ethical policy reasons, since this therapy includes a live medicine. In comparison, in Eastern European countries and Switzerland, phage therapy has been widely used for trials and therapies for long time [26,27]. Critical studies on phage therapy highlight either the eventual risks for humans and environment, or the efficiency level of the therapy. Here we report three works, which highlight the guidelines for the commercial phage cocktail (pyophage) certification. The high efficiency of treatments is continuously enriched by pinpointing the weakness of the experienced phage therapies [28,29,30]. 

An example of a restriction on the use of phage therapy could be a hazardous use of engineered constructs where a possible insertion could be translocated by a recombination to bacterial genomes that would acquire functions noncompatible with the environment, such as promoters or resistance genes. More studies should include and discuss the validity of those aspects as previously reported by the authors above. Most of the therapies were directed to overcome the effects such as inflammation, and production of metabolites rather than the principal causes [31]. 

## 2. Bacteriophages as a Therapeutic Strategy to Control Pathogenic Bacteria

Bacteriophages are viruses that infect, parasitize and kill bacteria. They interact with them through the specific recognition of receptor proteins of the bacterial surface. Bacteriophages are mainly double stranded DNA viruses classified in the order of *Caudovirales* with the families of *Myoviridae*, *Siphoviridae* and *Podoviridae* [32]. *Leviviridae* is a family of single stranded RNA viruses whereas the family of *Microviridae* is single stranded DNA [33]. Phages can be categorized in at least two subclasses including lytic (or virulent) and temperate (or prophage-lysogenic) phages. After interaction with their host, the DNA of the lytic phage is injected in the cell. Expression of its virulence genes induces the hijacking of the cellular machinery to exclusively replicate phage DNA and produce viral proteins. This viral cycle is characterized by the production of high numbers of viral particles within the bacterial cell leading to its short-term lysis. Filamentous phages with a single helix DNA can replicate and produce phages through extrusion. They present no lytic effect for the host cells. Several applications in molecular biology have been performed [34]. In contrast, DNA from a lysogenic phage can integrate in the bacterial genome in a dormant state known as prophage. Change of bacterial host environmental conditions can induce its specific replication eventually leading to a lytic cycle. Pseudolysogeny gives an additional alternative means of survival for phages that infect bacteria in nonoptimal conditions and survive in a plasmidic circular form after cell division [35].

Bacteriophages are naturally present in the microbiome and play an important role in maintaining bacterial community balance [29,30,31,32,33,34,36,37,38,39,40,41]. Sequenced bacterial genomes contain sixty to seventy percent of prophages [42]. Analysis of the human phage microbiome from fecal samples showed that prophages make up approximately 28% of all phages [43]. 

The new approach to gut microbiome study is derived from the discovery of extended interactions among its elements (virus, bacteria, bacteriophage etc.), which can lead to disequilibrium or health-equilibrium [44]. The study of microbiota should include analysis of phage-bacteria equilibrium and immune-response versus tolerance or disequilibrium with inflammation [45]. The spectrum of infection of one phage is usually narrow and limited to only a few strains of the same bacterial species. Thus, they do not change beneficial natural microbiota when compared to broad-spectrum antibiotics, but can eradicate undesired specific bacteria. 

The intestinal mucosa microenvironment has the highest amount of interaction between phages and bacteria because the mucin component of the mucus can capture phages and bacteria due to the polymeric structure of mucin consisting of glycans that are similar to immunoglobin like proteins that can bind the Hoc protein of the phage capsule [46,47].

The studies in vivo on the interactions between bacteriophages and gut microbiota have changed our vision when considering the life cycle, replication and survival of phages [48]. Several authors mentioned a possible role of phages in the maintenance of mucosal inflammation in Inflammatory Bowel Diseases (IBD) [49,50]. Hence, the phage population in IBD patients differs from healthy persons. Crohn’s disease patients present a lower diversity of bacteriophages compared to healthy patients [51] and an increase of phage quantity in the mucosa compared to healthy patients [52]. The authors should mention that further studies should be done to corroborate these findings. Although the recent studies are interesting and promising, more data are required in order to generalize results. In addition, the ulcerated mucosa presents a lower phage number than an unaffected one. Mechanisms that explain how phages regulate the bacterial flora in IBD or in healthy patients are still unclear. Some authors suggest that phage regulates the microbiota in a host-predator manner [45]. This is supported by the fact that Crohn’s disease patients have a higher number of bacteriophages but associated with a lower diversity. In addition, the transduction of bacterial genes by phages is a major mechanism of lateral gene transfer in bacteria. The prophage DNA induced during the lytic phase can include endogenic bacterial genes. This is particularly the case for antibiotic resistance genes and other bacterial virulence genes. Such a mechanism may help to decrease bacterial diversity and favor chronic inflammation. 

Additionally, highly lytic phages induce destruction of bacterial strains. This destruction in the mucosa increases the amount of released bacterial proteins that can be recognized by PRR system, and then potentially increase the risk of inflammation. These data support a model in which uncontrolled changes in the bacteriophage composition of microbiome may contribute to intestinal inflammation and bacterial dysbiosis [41].

Several studies propose a phage treatment as alternative and/or adjuvant to antibiotic treatment to control a bacterial infection in medicine [53,54,55,56,57,58,59,60,61] or agriculture [62]. Indeed, the use of widespread antibiotics has contributed to the global spread of resistant bacterial pathogens.

## 3. Phage Therapy to Modulate Microbiota Composition and Diversity

Among the perspectives on therapeutic modulation, the use of phage to manipulate bacterial population of the microbiota is highly interesting [63,64]. Phage therapy is applied either for rebalancing the microbiota in chronic diseases or for compassionate therapies in acute cases. In both cases the advantage of phage therapies with cocktails to reduce the risk of resistance is well recognized. Two strategies are used to enrich phage cocktails: (i) training the phages with a selection of local bacteria or (ii) tailoring the phage cocktail selecting the ones “trained” to infect the resistant bacteria as shown in intensive care patients [58,65]. 

The use of lytic phages has been proven efficient to reduce the number of pathogenic bacteria. Although such strategy implies that pathogenic bacteria are identified as major contributors of chronic diseases like *Helicobacter pylori* in the stomach or *Clostridium difficile* in secondary infection. To overcome this problem, a cocktail of six different phages has been set up by a Russian laboratory. These phage cocktails were analyzed and tested for adverse effects and toxicity but no negative effects have been reported [66]. Alternatively, inoculation of multiple lytic phages can be administered with lower amounts of antibiotics. This strategy is based on the synergetic action of antibiotics and reduced the bacterial resistance to phages. For example, a higher efficiency of antibiotics against *Pseudomonas aeruginosa* biofilms has been observed, which was due to a synergic effect of antibiotics and phages. Maximum killing was reached when the phage treatment was applied before the use of antibiotics, which could be explained by the disaggregation of biofilm by the phage activity, and thus induced a higher diffusion of antibiotics [67].

Bacteria can be defended against prophages using the Clustered Regularly Interspaced Short Palindromic Repeat (CRISPR) system [68]. The CRISPR-associated nuclease 9 (Cas9) bacterial immune system cleaves bacteriophage and plasmid DNA and can be used to selectively cleave antibiotic gene resistance or other virulence gene in bacteria brought by phages. Using this technique, Bikard et al. showed that Cas9 re-programmed to target virulence genes, kills virulent, but not avirulent *Staphylococcus aureus* [69].

Finally, phage therapy can be used to positively modulate the microbiota population. The development of genetic tools can be used to genetically modify some phages used alone or in combination with probiotics as vectors for nutrient biosynthesis or degradation that could favor the host. Unbalanced microbiota displayed low bacterial diversity and could potentially increase the proportion of pathogenic bacteria that favor mucosal inflammation. Manipulation of the microbiota by FMT, probiotics or specific diet is currently used. Alternatively, lytic phages could be used to selectively reduce pathogenic bacteria (Figure 1). Prophages that carry biosynthesis genes of metabolites and positively regulate mucosal inflammation could be engineered to genetically modify commensal bacteria. For example, the positive effect of SCFA produced by commensal bacteria has been previously shown for the benefit of obese patients [70]. We can imagine increasing the capacity of commensal bacteria to synthetize SCFA and H2S using genetically modified phages, which could be used as a treatment for obesity and affected mitochondrial metabolism, respectively (Figure 1). 

As phage therapy and the CRISPR/Cas9 system develop as tools for microbiota manipulation, further investigations in vitro and clinical trials are required to ascertain the various effects of using such phage-based treatment. 

## 4. Conclusions

The use of bacteriophages in Europe, Australia, and America is still experimental [71], and raises questions for ethical and safety reasons [72,73,74,75,76]. Despite the discovery of the Bacteriophages 100 years ago, by F.W. Twort and Felix d’Herelle, the delay in the usefulness of this tool is appalling. We have to change the paradigm of phage therapeutic application. Moreover it is important to consider the microbial world with its complete environment. Indeed, all is in a fine balance between bacteriophages, and commensal and pathogen bacteria. The disruption of phage-bacteria equilibrium in the microbiome is the key point that influences the prevalence of one of the two phage-bacteria actors. Two recent works explained and clarified how the phage can react to the CRISPR bacterial attack and the necessity of neutralizing the bacterial defence [77,78]. The phage therapy is an attempt to reduce or eliminate one species of undesired bacteria. The gut microbiota disequilibrium leads to a pathologic situation and the phage therapy is considered an efficient tool to establish a new equilibrium of the microbiota. The use of engineered phages can allow the elimination or reduction of bacterial defence. The digestion of bacteria biofilm could be an additional tool for increasing interest in the phage therapy [67].

New data concerning the role of the phages on the modulation of gut bacterial community are required to better understand the potential effect of phages on microbiota. The current literature lacks evidence of the detrimental effects of phages on the human immune system and the long-term effects of such human-bacteriophage interaction. Hence, some data suggests that phages can also stimulate immunity due to the coat protein of bacteriophages [79]. Moreover, the majority of phages and their specific spectrum are unknown. Thus, before treating patients with engineered prophages or lytic phages, further investigations are required for a better targeting of bacteria by phages. To overcome this problem a rigorous gut microbiota profiling must be part of the phenotypic analysis of chronic diseases for the most effective treatment. Despite the scarce information concerning each phage therapy, the microbiota targeting by phages is a new interesting strategy to change microbiota quality and diversity in the treatment of different pathologies related to disequilibrium of gut microbiota. It must be taken into consideration that with any microbiota transplant an important number of phages will inevitably be transplanted [47]. 

We are at the era of a deep transformation in our understanding of many diseases. We failed to combat many of them (obesity, Alzheimer’s disease, depression, etc.) with many pharmaceutical drugs, because we are still keeping our classical and static thinking. We need to change our vision and redesign a multitarget strategy, which can modulate microbiota diversity and mitochondria dynamics. Phages could be one of these actors.
